# Dermatoskopie des Melanozytoms – Neues oder Bekanntes?

**DOI:** 10.1007/s00105-024-05423-7

**Published:** 2024-10-21

**Authors:** Teresa Kränke, Andreas Blum

**Affiliations:** 1 Universitätsklinik für Dermatologie und Venerologie, Medizinische Universität Graz, Graz, Österreich; 2 Hautarzt- und Lehrpraxis Konstanz, Konstanz, Deutschland

Eine 56-jährige Patientin stellte sich im Rahmen der Hautkrebsvorsorge vor. Sie hatte keine neuen und/oder veränderten Läsionen bemerkt. Bei der klinischen Untersuchung fiel am linken Oberschenkel eine etwa 6 × 6 mm messende dunkelbraune Papel auf (Abb. 1a). In der Dermatoskopie zeigten sich ein großes zentrales dunkelbraunes Areal mit exzentrischer Aufhellung an 12–15 Uhr sowie ein mittelbrauner homogener Ring um diese Läsion (Abb. 1b).

**Abb. 1:**
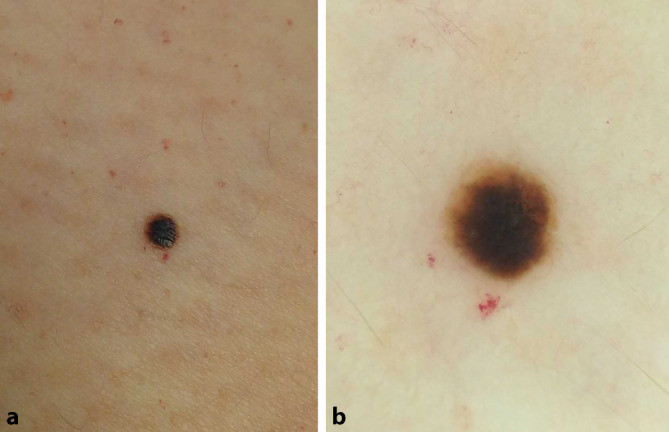
**a** Klinisches Bild einer dunkelbraunen scharf begrenzten Papel. **b** Dermatoskopisch zeigen sich ein großes zentrales strukturlos-dunkelbraunes Areal mit exzentrischer Aufhellung an 12–15 Uhr sowie ein mittelbrauner homogener Ring um diese Läsion

Welches therapeutische Management wählen Sie?

- Nach Hause schicken
- Kontrolle in 2 bis 3 Monaten
- Operation; wenn ja, mit welcher klinisch-dermatoskopischen Verdachtsdiagnose?

Die Läsion wurde mit dem Verdacht auf ein frühes Melanom exzidiert. Der histologische Befund ergab ein High-grade-Melanozytom.

Auf Basis zahlreicher histopathologischer, molekulargenetischer und immunhistochemischer Erkenntnisse scheint es neben der „klassischen“ dichotomen Einteilung melanozytärer Tumoren (Nävus vs. Melanom) noch eine dritte „Grauzonen“-Gruppe zu geben, die unter „atypische melanozytäre Neoplasien“ mehrere Entitäten zusammenfasst [1–9].

Da es weder klinisch noch histopathologisch einheitliche Kriterien für diese Gruppe gibt und sie sich zudem deutlich in ihrem immunhistochemischen Profil sowie in ihrer Biologie unterscheiden, wurde von der WHO(Weltgesundheitsorganisation)-Arbeitsgruppe folgende deskriptive Terminologie respektive Einteilung für die histopathologische Diagnostik vorgeschlagen [3]:

1. intraepidermale atypische melanozytäre Proliferation von unklarem malignem Potenzial (IAMPUS): Ähnlichkeiten zu In-situ-Melanomen,
2. superfizielle atypische melanozytäre Proliferation von unklarem malignem Potenzial (SAMPUS): dünne melanozytäre Neoplasie mit Compound-Komponente, die Ähnlichkeiten zu frühinvasiven Melanomformen zeigt,
3. melanozytäre Tumoren mit unklarem malignem Potenzial (MELTUMP): Neoplasien mit einer Compound-Komponente oder intradermal gelegenen Zellen, sodass sie nodulären Melanomen ähneln.

Der Vorteil dieser Einteilung ist, dass hier auch Melanomsimulatoren wie „atypische Nävi“ und biologisch „intermediäre“ Tumoren (melanozytäre Tumoren mit niedrigem malignem Potenzial) eingeschlossen werden [3].

Die MELTUMP gehören der histopathologischen Gruppe der „atypischen intradermalen ‚kanzerogenen‘ melanozytären Neoplasien“ („atypical dermal-based ‚tumorigenic‘ melanocytic neoplasm“) an. Hierzu zählen unter anderem auch die Melanozytome und Spitz-Tumoren.

Melanozytome werden wie folgt untergliedert:

- BAP1-inaktiviertes Melanozytom,
- Deep-penetrating-nevus-like-Melanozytom (wie in unserem Fall),
- pigmentiertes epitheloides Melanozytom.

Das BAP1-inaktivierte Melanozytom wird je nach weiteren Kriterien als Low- oder High-grade-Melanozytom klassifiziert, während das pigmentierte epitheloide Melanozytom per definitionem als „high-grade“ eingestuft wird. Das BAP1-inaktivierte Melanozytom wurde 2011 erstmals von Wiesner et al. [4] in 2 unabhängigen Familien einer Keimbahnmutation im Tumorsuppressorgen BRCA1-assoziiertes Protein 1 oder BAP1 beschrieben. Einzelne dieser Tumoren können sporadisch auftreten, wohingegen multiple Läsionen typischerweise auf unterschiedliche familiäre Tumorsyndrome hinweisen. Das Deep-penetrating-nevus-like-Melanozytom ist in aller Regel biologisch gutartig und hat eine gute Prognose. Es gibt vereinzelt Fallberichte von „Borderline-Tumoren“, wo es zu Absiedelungen in die regionalen Lymphknoten gekommen ist.

## Klinik und Dermatoskopie

Klinisch imponieren Melanozytome in der Regel als einzeln stehende, stark pigmentierte rötlich-dunkelbraune, selten auch blaue Papeln oder Knoten. Die Prädilektionsstellen sind die Extremitäten und der Kopf‑/Hals-Bereich. In seltenen Fällen kann es zum Auftreten von multiplen Melanozytomen bzw. Clustern von Melanozytomen kommen. Einige der Läsionen liegen dann gruppiert zusammen (aggregierter Typ) und können nach Entfernung einer Läsion rezidivieren.

Treten mehrere Tumoren auf, erinnert das klinische Bild an ein Melanom mit Satellitenmetastasen; eine klinische und auch dermatoskopische Unterscheidung ist nahezu unmöglich, sodass eine histopathologische Beurteilung zwingend erforderlich ist [1, 3].

Zur Dermatoskopie von Melanozytomen gibt es wenige Daten [7, 8]. Ein rezenter systematischer Review [7] konnte in der Literatur insgesamt 16 Fälle von Melanozytomen und deren dermatoskopischen Mustern finden. Alle Läsionen zeigten zentrale, sehr große, homogene oder strukturlose dunkelbraune oder blaue Areale. Bei der Hälfte der Fälle traten zusätzlich schwarze Areale auf und in 6 Fällen außerdem weiße Linien („shiny white lines/crystalline structures“) auf. Eine weitere Arbeit [8] über BAP1 inaktivierte melanozytäre Tumoren wies nach, dass diese Läsionen klinisch auch als kuppelförmige rosafarbene Papeln imponieren können. In der Dermatoskopie konnten 5 vorherrschende Muster identifiziert werden, wovon folgende ausschließlich bei BAP1-inaktivierten melanozytären Tumoren (im Vergleich zur Kontrollgruppe) gefunden wurden: strukturloses Areal mit exzentrisch gelegenen Punkten/Globuli und retikuläres Netzwerk mit strukturlosen erhabenen Arealen.

## Histopathologie und Immunhistochemie

Die histologische Unterscheidung zwischen einem schwer atypischen Melanozytom und einem Melanozytom-ähnlichen Melanom stellt ein großes Problem dar. Eine Reihe von architektonischen (etwa Durchmesser, Asymmetrie, Ulzeration) und zytologischen (z. B. mitotische Aktivität und Tumornekrose) Kriterien kann bei der Diagnose helfen. Zudem sind einige immunhistochemische und molekulargenetische Marker bekannt, um eine Differenzierung zu ermöglichen.

## Exzision

Die vollständige Exzision sollte mit einem entsprechenden Sicherheitsabstand – je nach Risikoprofil mit 5–10 mm – erfolgen und dies stets mit einer randkontrollierten Histologie ohne atypische Melanozyten [3, 9].

## Wie sollen wir unsere Patienten nachbetreuen?

Derzeit existieren keine einheitlichen Richtlinien, aber praktische Empfehlungen unter anderem von der EORTC (European Organisation for Research and Treatment of Cancer) [9].

Eine hautfachärztliche klinisch-dermatoskopische Untersuchung mit besonderer Berücksichtigung der Narbenregion und Palpation der drainierenden Lymphabflusswege alle 12 Monate ist unserer Meinung nach heute ausreichend und dem biologischen Verhalten von Melanozytomen angepasst. Staginguntersuchungen wie etwa eine Sonographie der Lymphknoten könnte nur bei High-grade-Melanozytomen halbjährlich für 3 Jahre sinnvoll sein [9].

## Schlussfolgerung

Wichtig ist, Melanozytome zu erkennen respektive daran zu denken bzw. bei Läsionen, die man nicht sicher zuordnen kann und die zudem noch knotig sind (wie in unserem Fall), die Indikation für eine Operation großzügig zu stellen. Die randkontrollierte Histologie sollte stets frei von atypischen Melanozyten sein. Die Nachsorge sollte engmaschig, aber pragmatisch – ähnlich der einer schweren melanozytären Dysplasie – erfolgen.
